# Patient Experiences in a Linguistically Diverse Safety Net Primary Care Setting: Qualitative Study

**DOI:** 10.2196/jopm.9229

**Published:** 2018-01-22

**Authors:** Rachel L Berkowitz, Nimeka Phillip, Lyn Berry, Irene H Yen

**Affiliations:** ^1^ DrPH Program School of Public Health University of California, Berkeley Berkeley, CA United States; ^2^ Residency Program Mountain Area Health Education Center Hendersonville, NC United States; ^3^ Division of Primary Care Department of Medicine Alameda Health System Oakland, CA United States; ^4^ Department of Medicine University of California, San Francisco San Francisco, CA United States; ^5^ Department of Epidemiology & Biostatistics University of California, San Francisco San Francisco, CA United States

**Keywords:** safety-net providers, urban health services, primary health care, patient-centered care, qualitative research, quality of health care, communication barriers

## Abstract

**Background:**

The patient-centered medical home model intends to improve patient experience and primary care quality. Within an urban safety net setting in Northern California, United States, these desired outcomes are complicated by both the diversity of the patient community and the care continuity implications of a residency program.

**Objective:**

The objective of our study was to understand the patient experience beyond standardized satisfaction measures.

**Methods:**

We conducted a qualitative study, interviewing 19 patients from the clinic (English-, Spanish-, or Mien-speaking patients).

**Results:**

Some themes, such as the desire to feel confident in their doctor, emerged across language groups, pointing to institutional challenges. Other themes, such as distrust in care being provided, were tied distinctly to speaking a language different from one’s provider. Still other themes, such as a sense of powerlessness, were related to cultural differences and to speaking a language (Mien) not spoken by staff.

**Conclusions:**

Findings illuminate the need to understand cultural behaviors and interactional styles in a diverse patient population to create a high-quality medical home.

## Introduction

I don’t know because I don’t understand English, and then whatever I tell the interpreter, he is relaying the information in English, and then relaying what the doctor says back to me. There is a gap.

Mien-speaking patient

This comment was shared by a Mien-speaking patient at the Lakeview Hospital Adult Medicine Clinic (LHAMC, located in an urban setting in Northern California, USA; we have changed the name of the hospital to protect the privacy of the patients) and provides a window into one of the challenges experienced by non-English-speaking patients within a multilingual, multicultural urban safety net setting.

Patient-centered care is an increasingly promoted approach for improving quality of, access to, and satisfaction with health care, particularly for primary care [1,2]. To fully implement patient-centered care, we must partner with patients and understand how to strengthen their experiences and outcomes [3]. Many factors shape the way patients perceive quality of care. In urban safety net clinics, understanding those experiences can be challenging. Different constructs of health, levels of acculturation, and institutional barriers implore clinicians to find new methods to explore patient experiences [4]. When patient views are solicited through qualitative methods, results emphasize aspects of the patient experience that may have otherwise gone unnoticed and could effectively shape patient experience, clinical functioning, and patient outcomes [5].

At the time of this study, the LHAMC was beginning the conversion to a patient-centered medical home (PCMH) model of care. The proposed operational changes required that clinic leadership better understand the views of patients in order to incorporate specific interventions that would increase patient satisfaction. The conversion also brought the opportunity to explore some of the barriers to patient satisfaction, such as the transient nature of resident physicians providing primary care and language barriers faced by the patients, who speak over 35 different languages.

The presence of resident physicians may contribute to fragmented care and other negative patient experiences that compound the challenge of providing high-quality patient-centered care. At the time of this study, approximately 50% of the clinic’s patients received their care from resident physicians in an internal medicine training program that used the traditional curricular model of a weekly continuity session during all rotations. Residents can positively influence quality of care and outcomes by developing close interpersonal relationships with patients [6]. Conversely, when a resident cares for patients with low English proficiency, misuse or underuse of interpreter services by the resident may negatively affect the patient’s perception of quality of care [7].

Language barriers can also contribute significantly to differences in patient satisfaction. Patient-provider language discordance may negatively influence patient experience, leading to the patient disclosing less information and feeling negatively judged, vulnerable, disrespected, and helpless [8]. Furthermore, in language-discordant provider-patient interactions, time constraints, availability of interpreter services, and other institutional challenges can lead to disparities in the quality of the communication experience, patient satisfaction, and patient health outcomes [9-12]. To help understand the impact of language on the patient experience at LHAMC, we applied the concept of *cultural health capital* (CHC). The CHC framework “provides a way to understand how features of patient-provider interactions—such as interpersonal rapport, exchange of information, empathy, and trust—are accomplished or undone, based upon the repertoire of specialized cultural resources that patients bring to the health care encounter, in combination with providers’ fostering of and receptiveness to those resources” [13]. This theoretical model was important in analyzing the clinical interactions described by the LHAMC’s patients.

The Building Together Project began in January 2014. As the LHAMC continued its process of converting to a PCMH, staff and researchers recognized the need to engage directly with members of the urban safety net clinic’s diverse patient community. A team of 1 attending physician (LB), 1 resident, 2 medical interpreters, 1 researcher (RLB), and 1 English-speaking patient came together with the goal to use qualitative research methods to explore the following research questions: (1) What makes a clinic experience for a patient positive, and why? (2) What makes a clinic experience for a patient negative, and why? (3) How do patients’ personal, cultural, and historic contexts affect their clinic experience? By gaining a deeper understanding of diverse patient experiences within the LHAMC, we hoped to provide a more nuanced foundation on which to build ongoing patient-centered quality improvement.

## Methods

### Setting

Lakeview Hospital is part of a county health system and sees over 180,000 outpatient visits a year, serving patients who speak over 35 languages. The LHAMC is a primary care medical home for adult patients within the hospital, seeing approximately 8000 patients a year for preventive, acute, and chronic care. Over 80% of patients use Medicaid, and 15% use the county health program for uninsured residents.

We focused on 3 patient language groups: English-speaking, Spanish-speaking, and Mien-speaking patients. English and Spanish were the 2 most commonly spoken languages within the LHAMC, listed as the primary language spoken for 73% and 18% of patients, respectively, in fiscal year 2013. Speakers of Mien, who originate from southern China and northern Southeast Asia, and who made the largest percentage of recorded LHAMC interpreter requests in fiscal year 2013 (29% of requests), are not often able to participate in English- or Spanish-language feedback opportunities. Mien is a traditionally oral language, which could lead to unique challenges in navigating the hospital system.

The Building Together Project study procedures were approved by the health system’s institutional review board (IRB14-02041A).

### Interview Guide

**Figure 1:**
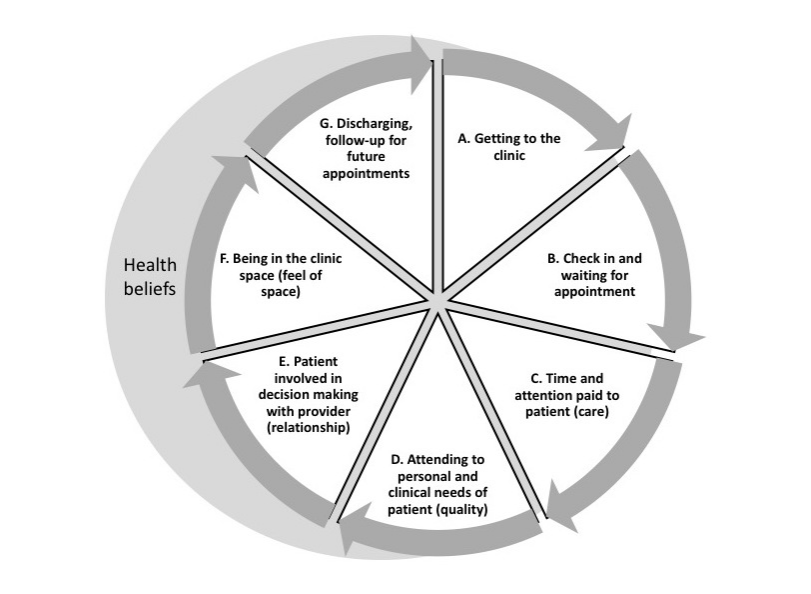
Patient experience wheel for a multilingual, multicultural urban safety net clinic.

To ensure that the interview guides and protocol reflected the experiences and perspectives of the diverse members of the team, the team’s researcher member facilitated an experiential training on qualitative research and interview guide development with team members. After discussing the subjective paradigm and text-based data of qualitative research [14], the team reviewed the structure and function of an interview guide and reflected on the 3 driving research questions of the project. The team then developed our own clinic patient experience wheel to identify the aspects of the patient’s experience that they wanted to ensure could be explored, based on an example from Western Australia [15]. The wheel shows the different experiences that patients move through as a part of a visit to the clinic, beginning with getting to the clinic and concluding with discharge and follow-up for future appointments, which leads back to the first step (see Figure 1).

After creating the wheel, the team identified 3 overarching aspects of patient experience: (1) space and procedures of the clinic, (2) relationships with staff in the clinic, and (3) experience of care and beliefs about health. These 3 aspects became the topical frames for the study’s interview guide. The team composed questions, ensuring that the language would translate well from English into Spanish and Mien.

### Interview

Patients were recruited in the waiting room of the LHAMC. The researcher along with the team’s interpreters conducted face-to-face interviews in a room nearby but outside of the clinic so as not to conflate the interview experience with the patient’s clinic visit. We set the goal of interviewing 16 to 24 patients (8-10 English-language, 4-6 Spanish-language, and 4-6 Mien-language interviews) based on resource and time constraints, as well as the hope of achieving thematic saturation related to language- and culture-specific issues within each group [16].

All interviews were conducted in English (with support from a Spanish-speaking or a Mien-speaking interpreter for those language-specific interviews). After each interview, the interviewer and interpreter had a debriefing conversation, and the interviewer recorded notes if shifts or changes were made to the interview guide or procedure. Following the first interviews in each language group, the team made additional minor changes to the structure and wording of questions. Most notably, the team shifted the Mien-speaking patient interview guide to accommodate the refusal of Mien-speaking patients to have their voice recorded, either for fear of ramifications or a belief that recording one’s voice could ultimately trap one’s soul. Accordingly, the Mien-speaking patient interview guide provided space for the researcher to write notes to record Mien-speaking patient responses to questions and probes. Patients received a US $20 gift card for completing the interview.

### Analysis

This study used thematic analysis, a methodology that manifests differently depending on specific parameters set by a study’s researchers, emphasizing the importance of transparency on the part of researchers in articulating the assumptions, decisions, and actions that lead to the ultimate analysis of themes [17]. Of priority was incorporating the team’s diverse perspectives into the development of codes and analysis of transcripts, drawing on the principles of community-based participatory research, in which the inclusion of nonacademic researchers connected to the circumstances of interest throughout the research process enhances the investigation and findings [18].

The recorded interviews from the Spanish-speaking and English-speaking patients were transcribed throughout data collection by the researcher and a transcription assistant. Notes from Mien-speaking patient interviews were typed up by the researcher as soon as possible following the interview to capture as many of the Mien-speaking patient’s own words as possible.

#### Coding

The team used an adapted consensus-building approach with a sample of 6 representative transcripts (2 from each language group) to develop a codebook that could be applied to the remaining transcripts. First, all participating team members applied the coding steps to the same part of one transcript. The group compared findings, looking for similarities and differences, ultimately coming to an agreement about the overarching codes. Next, 2 pairs of 2 team members were given 2 transcripts or notes from patients of different language groups to code. Within the pairs, each individual completed the coding steps independently and then discussed the identified codes with his or her coding partner, ultimately coming to a consensus around the codes present within the transcript (or notes).

Codes and definitions were sorted and grouped into similar categories. This process resulted in 8 overarching codes: (1) continuity of care, switching doctors, and resident turnover; (2) health care system, structure, and navigation; (3) relationship and communication between staff, health care team, and patient; (4) access to and experience with interpreters; (5) language and culture differences or similarities; (6) experience of waiting; (7) what it means to be healthy or unhealthy; (8) perspectives on needed improvements in the clinic.

The researcher, attending physician, and patient next independently coded the remaining transcripts using this codebook. Finally, the researcher reviewed all coded transcripts and notes to ensure that all aspects of the interviews relating to the 8 codes were captured.

#### Thematic Analysis

The researcher and attending physician reviewed coded segments within specific codes to identify themes relevant to answer the research questions. The team reviewed these themes to ground the findings in the team members’ unique experiences and perspectives. The researcher then looked across all codes, comparing ideas within and between language groups to identify broader patterns of significance in relation to the initial research questions.

## Results

### Patient Demographics

Between February and May 2014, the team conducted 19 interviews—8 in-person and 11 over the telephone—with recordings ranging from 13 to 60 minutes. Table 1 presents the demographic characteristics of the participants. English-speaking patient participants were younger, more likely to be born in the United States, and more likely to have some education than were the Spanish-speaking and Mien-speaking patients.

### Common Themes Across all 3 Language Groups

#### A Personal Relationship With a Provider—Feeling Cared For

Patients from all 3 language groups described what made a good doctor-patient or staff-patient relationship, including the desire to feel confident in their doctor, and to have their doctor listen to them and engage with them, explain treatments and options, and show concern for their well-being.

[An ideal interaction with a doctor] would be one on one. And also that the doctor or practitioner would engage me too. To see you in the eye to see you as a person and not just a statistic.

English-speaking patient

**Table 1:** Interview patient demographics.

Characteristics		All patients (N=19)	English-speaking patients (n=8)	Mien-speaking patients (n=6)	Spanish-speaking patients (n=5)
Age (years), mean		55	46	67	56
**Country of birth, n**					
	United States	7	7		
	Mexico	6	1		5
	Laos	5		5	
	China	1		1	
**Education, n**					
	Some	14	8	2	4
	None	5		4	1
**Work, n**					
	Not working	16	5	6	5
	Working	3	3		
Female sex^a^, n		11	5	3	3

^a^Sex was defined by the interviewer and not by the patient.

#### “Knowing My History”

Within reflections on relationship, patients emphasized the importance of their doctor knowing their history, and the frustration experienced when a doctor asked repetitive questions or clearly suggested in other ways that the doctor was unaware of the patient’s history, wasting the limited time of the visit.

I feel like it’s not right for me to start talking again, and again and again every time I go to see a doctor and every time I been seen by someone new to start talking about my old medical issues, my old problems. So I want them to focus on what I am telling them at the moment but they just keep asking me question about the old stuff.

Spanish-speaking patient

#### The Challenge of the Resident Doctor

Patients discussed the impact of having a resident as their doctor, including feeling that the doctor was just using them for training and would abandon them, feeling confused as to why doctors left after their training, or feeling as if their doctor was not a “real” doctor yet.

I believe that they are not a real doctor...I feel like they just use me as an object for their training...If he is my doctor, he is always there to help me, he knows what I need, he is there to help me. But with the residents, it’s like a girlfriend and boyfriend—if I like you, I’ll stay for a little. If I don’t I’ll leave...if they don’t like caring for you, they go away.

Mien-speaking patient

#### Communication Issues Within Lakeview System

Patients described challenges with understanding what and why changes were happening at the clinic level (such as a delay getting an appointment), and with their ability to speak directly with doctors about issues. Patients also described challenges with the LHAMC communicating with other clinics within the hospital system to coordinate care or to follow up on tests or treatments needed.

Experiences varied, however, as some patients offered examples of good-quality communication experiences.

Well, I from the very beginning, I notice even when I start getting to the desk information. I notice that the information given to me was very clear...at Lakeview there are signs where you need to go to ask for information and that makes the, um, services to be better, more effective, and what you need to do.

Spanish-speaking patient

#### Waiting

Across language groups, patients discussed the frustration with waiting to be seen during their appointment at the clinic, waiting to get an appointment, or waiting in other contexts (including the pharmacy) within the hospital.

[I]t’s just very its frustrating because they are so slow its just so slow it takes long times to be called up there to register and then you have to finally get registered which is gunna be after your appointment and even though I come early it takes them so long to register to call you up to register you that they call you after your appointment. But it doesn’t matter because the doctor is gunna take forever (laughing) to see you so umm it’s just really frustrating that’s what I hate about Lakeview the waiting the slowness that and that’s for the whole hospital for everything. Everything is just slow.

English-speaking patient

### Themes Related to the Added Impact of Language

The Mien-speaking and Spanish-speaking patients highlighted experiences and realities, not described by the English-speaking patients, that were particular to the realities of speaking a language other than English within the health care system. Within these themes, the experiences of Mien-speaking and Spanish-speaking patients also differed based on the commonality of each language among clinic staff.

#### Impact of Language on Relationships

Mien-speaking and Spanish-speaking patients emphasized the added impact of language differences and commonalities on establishing trust and a good relationship between patient and provider and feeling confident that the patient’s needs were being understood.

Maybe I don’t speak English, they treat me differently. I look at their actions. Maybe with English-speaking patients they change and act differently.

Mien-speaking patient

[Seeing a doctor who does not speak Spanish] makes the visit to the doctor’s more difficult because we don’t have any clear communication. Maybe the symptoms will be, uhh, not interpreted correctly.

Spanish-speaking patient

Some Mien-speaking patients also suggested that not speaking the same language as clinic staff or providers could have an impact on their overarching experience in moving through the appointment process.

Waiting [in the waiting area] is also hard because I have to wait a long time, sometimes a half hour, sometimes more, one and a half hours. For me, I don’t have to wait long but my mom sometimes waits all day. I suspect because she does not speak English and cannot push at registration.

Mien-speaking patient

Mien-speaking and Spanish-speaking patients also talked about other ways in which providers reached across language barriers to establish trusting relationships, including an emphasis on tone and touch (Mien), trying to speak some Spanish or identifying a new provider for a patient who did speak the same language (Spanish), and insisting to the interpreter that all of the information that the patient wanted to share was important to the doctor (Spanish).

[My] doctor is good. She understands, the way she talks is very caring. She is not like other doctors, other doctors don’t have time to listen to you, and then their tone of voice is harsh. She understands culturally and she takes time to listen to a patient.

Mien-speaking patient

And he was always very kind, always trying to help me but every time I saw him, that was with the help of an interpreter. And I think that he understood a lot because sometimes when I was telling the interpreter there’s some things the interpreter used to tell me, “Well that’s not important for the doctor to know that” or “that is not something that the doctor can help you with that,” so and I was just telling the interpreter and many times the doctor say, “What, what they are telling me? No, no, tell me. What is what she wants? Tell me what is what she saying?” And the interpreter used to say, “Well it’s not something that is important for you, doctor. That is an area different to yours.” And he always say, “No, I wanna know. I want to know anything related to the patient.”

Spanish-speaking patient

#### Role of the Interpreter

Mien-speaking and Spanish-speaking patients discussed the role of the interpreter as a facilitator of conversation and care between doctor and patient. While many interpreter interactions described by patients referred to in-person interpretation, some patients did describe the use of the video interpreting system. Most of these patients found that experience to be positive, with one Mien-speaking patient stating explicitly that face-to-face interpretation was preferred. Most patients saw having an interpreter as a positive and vital part of the relationship (and insisting on having an interpreter present was seen as key).

My problem is that I don’t speak English, but when I see a doctor, there are interpreters that help me through the machine, and so that is good, that is very helpful for me. And I don’t have any problem with that. And what I see is that everybody who goes there, there is no distinction, there is no discrimination, everybody is being seen in the same way and treated in the same way, so that is good for me.

Spanish-speaking patient

I would like to see somebody who pays attention to me, who can hear my concerns, who can communicate with me better, who don’t assume that I speak English. My medical terminology is not so good, so get an interpreter for me.

Mien-speaking patient

Other patients emphasized the potential for things to be lost in the conversation or not feeling confident that the interpreter was adequately conveying the needs of the patient, which was detrimental to the relationship. The power held by the interpreter as the arbiter of conversation was highlighted as potentially problematic.

#### Waiting to Speak

Spanish-speaking and Mien-speaking patients discussed the additional frustration of waiting just to speak—waiting to have an interpreter available (in person or through the video interpreting system) and then waiting during the visit itself while the conversation was being translated back and forth. Waiting for an interpreter was particularly frustrating, given how little time patients felt they had with the doctor during an appointment. Patients also discussed this adding to a sense of feeling bad because they did not speak English.

[S]ometimes there is a little problem because the nurses do not speak Spanish...and sometimes they need to call or bring someone who speaks the language and sometimes they cannot find it... [so] they try to look for someone to come in an interpreter, or they can, they look for someone who is nearby next to me who can speak Spanish so they use the person... [and] it makes me feel a little bit bad because I do not understand English.

Spanish-speaking patient

[Explaining why it would be better to just use a family member as an interpreter] The reason is if it’s not difficult, then you can use the family member, hurry, finish up, and go home. Because the interpreter may be busy and you may have to wait longer. Yeah, wait a very long time, sometimes they have to finish what they are doing.

Mien-speaking patient

## Discussion

### Principal Findings

Across the 3 language groups, patients emphasized the importance of a good-quality relationship with their doctor and staff. They highlighted the importance of empathetic listening, supportive explanations of health issues and treatments, and a demonstration of understanding a patient’s history during a visit. Qualitative and quantitative studies alike emphasize the importance to patients of clear and positive communication with providers (and ancillary staff) in which providers and staff listen to their patients, show concern for their well-being, and spend time to clearly explain health issues, treatment options, and other procedural realities [3,8,19].

Patients described the unique challenges of having a resident as one’s primary care physician and engaging with a residency training program overall, emphasizing in particular the challenges with abrupt and at times unexplained discontinuity with resident providers, as well as a negative feeling of being “trained on” and not being seen by a “real” doctor. Studies provide evidence of the importance of provider continuity for patient satisfaction, reduced emergency medical use, and even some health outcomes [5,20]. Traditional residency training schedules can make it difficult for patients to achieve a sense of interpersonal or relational continuity (terms that describe long-term, trust-based relationships between providers and patients in which patients sense a provider’s commitment to the patient’s well-being) with their resident physicians [21]. In addition, residents receive limited training in the use of professional interpreter services and may underuse these resources, particularly if they have some proficiency in a patient’s language or if a patient brings a lay interpreter such as a family member to a visit [22-24]. Since this study, LHAMC’s residency program was converted to a 3+1 curriculum in which residents spend 1 out of 4 weeks in the clinic without interference from any other clinical responsibilities. In addition, LHAMC has formed a team system in which each resident works closely with faculty, nurse practitioners, and nursing staff, who provide continuity during the 3 weeks when the resident is on another service. These changes appear to have resulted in improved patient satisfaction with resident physicians as continuity providers as measured by a recent survey of 200 sample patients. As measured by the annual US Accreditation Council for Graduate Medical Education survey, the residents have voiced a significant increase in their satisfaction with practicing primary care in the clinic.

For the Spanish-speaking and Mien-speaking patients, speaking a language other than English added another layer of complexity and difficulty regarding basic interaction with doctors and staff, as well as interaction with residents specifically. Patients described a general concern as to whether doctors and patients fully understood each other when having to work through an interpreter. Patients highlighted that doctors and staff did try to reach across the language barrier to establish positive relationships. For Spanish-speaking patients, however, identifying a Spanish-speaking provider was seen as ideal. For Mien-speaking patients, the option of identifying a language-concordant provider or staff members is not yet possible within the LHAMC, and this reality can lead to feeling lost in spaces such as the waiting room. Mien-speaking and Spanish-speaking patients alike emphasized the importance of having an interpreter available when language concordance with a provider is not an option. Patients also highlighted the frustration of waiting for an interpreter, waiting just to speak, particularly when appointment times are so truncated. Mien-speaking patients described wishing a family member could be used to speed up the waiting time. While the potential benefits of having a strong advocate for the patient serve as the interpreter are important, concerns regarding the accuracy of information being transferred are also important to consider [25,26].

Studies have found that having an interpreter can add time to the length of a patient visit, particularly in relation to the interaction with the provider [27]. While video- and telephone-based interpreting systems may decrease waiting times for interpretation, remote interpretation systems may not decrease the length of the visit itself or may not be preferred in relation to the visit quality [27,28]. In surveys, LHAMC physicians have also voiced the concern that patients who are not language concordant should be given the same amount of appointment time as patients who are language concordant with their physician. This either limits the quality of the visit or results in longer visits, increasing wait times for subsequent patients.

The added impact of a language difference on the patient experience of primary care, which is tied to feeling confident in developing a good-quality relationship with a provider, can be understood in the context of CHC [13]. CHC encompasses the various skills, cultural understandings, and attitudes that allow a patient to satisfactorily navigate the health care system and patient-provider interactions. Through the lens of CHC, lacking such competencies creates and perpetuates inequities within a health care setting.

Patient characteristics constituting CHC include “knowledge of medical topics and vocabulary,” as well as “the skills to communicate health-related information to providers” [13]. While difficulties with these characteristics are not limited to non-English-language speakers, the situations described by Mien-speaking and Spanish-speaking patients—having to either communicate in a second language or communicate through an intermediary—add another barrier to learning and using CHC to achieve better quality of care. All of the English-speaking patients had some education, and most were born in the United States, potentially enhancing the ability of these patients to navigate the predominantly English-speaking US health care system. As one Mien-speaking patient discussed, not speaking English also hindered self-efficacy related to engaging with the registration staff to understand the delay being experienced by the patient’s mother. This touches on another component of CHC—having “an enterprising disposition and a proactive stance toward health” and one’s care [13]. While CHC is something that one can cultivate over time through repeated interaction with providers and the health care system, when one does not speak the same language, it may be more difficult to gain a full embodiment of these characteristics. If there is a fundamental uncertainty as to whether a given encounter is being fully understood by a provider, how can a patient build the habits and instincts that can enhance the patient experience and quality of receiving care?

CHC is not limited to identifying the characteristics that patients need to effectively maneuver through a health care interaction. The CHC concept also emphasizes the interpretation of CHC characteristics by physicians, stating that the physician’s interpretation can affect how he or she unconsciously perceives and ultimately treats the patient. As Shim describes, “patients and family members who mobilize CHC to present themselves and their health issues in approval-garnering and medically intelligible ways can generate ‘cascades’ of subsequent interactions and actions...that may enhance communication and care” [13]. The opposite is also true. If a physician notices a patient’s lack of CHC characteristics—such as the ability to communicate effectively about medical circumstances—the physician may inadvertently alter the way that he or she provides care, giving an impression of impatience or lack of concern akin to what some patient participants in this study noted. As the patient participants described, feeling as if a doctor is not actively cultivating the patient-provider connection can have a detrimental effect on the patient experience.

### Limitations

Limitations of interview structure and time precluded our abilities to explore cultural perceptions of health and well-being in depth, which would have added to the understanding of diverse patient communities’ engagement with primary care, as well as the broader application of the CHC structure. Spanish-language and Mien-language interviews were conducted with support from interpreters; while the researcher and interpreters took care to ensure clear communication throughout the interview experience, there remains a potential for some information to have been lost or misconstrued in translation. Also important to note, the study could not encompass the full ethnic and linguistic diversity of the Spanish-speaking and English-speaking populations of LHAMC in the 13 interviews conducted for this study. Indeed, engaging with the full linguistic and ethnic diversity of the clinic was beyond the scope of this study. While themes were repeated within each language group, suggesting a degree of saturation in some thematic areas, additional interviews may have revealed further elements of the patient experience.

### Implications

The process of recruiting patients and conducting the interviews for this study was the basis for important patient-centered quality improvement efforts within LHAMC. Projects to date have specifically addressed the themes that emerged from these interviews. For example, having access to a provider who knows the patient’s medical history was addressed by dividing the staff into 4 care teams who cover for one another and share information routinely. Since this study, the clinic has adopted an electronic health record system and have implemented standard operating procedures, which require a previsit medical record review and huddle with the care team. Patient flow has improved and a waiting room protocol developed by the council was introduced to inform patients of the estimated wait times.

In addition, building on the lessons learned from the patient interviews, the clinic received external funding to develop a multisectoral, patient-centered primary care council. Since July 2014, this council, consisting of English-speaking, Spanish-speaking, and Mien-speaking patients, as well as clinic staff and medical providers, has met on a monthly basis to explore patient-identified challenges within the clinic environment and to develop pilot projects in partnership with the clinic to address those challenges. The standardized Clinician and Group Consumer Assessment of Healthcare Providers and Systems scores for our clinic have improved by approximately 12% per year over the 3 years of the council’s existence. The council has created a unique pathway through which the clinic can continue to engage with patient perspectives for enhancing the primary care experience. Over the past year, the council has been asked to comment on the development of quality improvement processes that have been mandated in our clinic through our safety net Medicaid waiver program. This includes the introduction of universal screening for depression and substance use, addressing our patients’ sexual orientation and gender identification, and developing the messages to protect and support our undocumented patients. The council feedback has been reported to our Board of Trustees and the health system administration, who have used many of their suggestions in developing these programs.

The research approach used in this study demonstrates the strength of engaging with diverse perspectives in developing, executing, and analyzing the results of a qualitative study. The study’s findings highlight the importance of hearing the patient perspective as a component of developing a PCMH. In particular, findings highlight the added impact of linguistic differences between patients and clinic providers and staff. Understanding the challenges experienced by linguistically and culturally diverse patient communities has important implications for medical practice and education. While the themes highlighted by patient participants have been touched on in the literature, additional qualitative and quantitative research is needed to develop pragmatic methods to address key issues such as provider-patient interactions, the differential experiences of non-English-speaking patients, and the added effects of residency training programs on patients. Translating such research into practice is equally vital. Already, curricula that incorporate recognition of language and cultural differences into residency training are described in the literature [29,30]. In addition, the preference for language concordance with providers among patients who speak a language other than English suggests the importance of supporting medical training for physicians and staff who speak languages other than English and of developing methods to support such providers to practice within linguistically diverse settings in the United States [31].

Shim highlights that low-resource health care settings are simultaneously “more likely...to serve patients who lack significant cultural skills” to navigate the health care setting and more likely to be subject to the constraints of resources and time that would allow providers to “help patients become better participants in their own care” [13]. This situation is certainly true of LHAMC, where the hectic, packed schedules of attending physicians and residents necessitate 20-minute patient visits; such constrained visits are made even briefer when an interpreter is involved, cutting the amount of actual communication in half. The potential benefits of intentionally supporting effective patient engagement with the health care system and the individual doctor-patient interaction—in terms of saving time, money, and health in the future—suggest the need for safety net settings such as LHAMC to lengthen the patient visit time, allowing for provider-patient relationships to deepen even when a third party is necessary to broker a language gap. Incorporating the patient perspective—beyond isolated results from overarching patient satisfaction survey data—into every aspect of the clinician’s role can enhance the patient’s ability to fully engage with a primary care visit, giving the patient his or her best chance to benefit from that experience.

## Abbreviations

CHC: cultural health capital
LHAMC: Lakeview Hospital Adult Medicine Clinic
PCMH: patient-centered medical home
